# LncRNA CASC11 promotes cancer cell proliferation in hepatocellular carcinoma by inhibiting miRNA-188-5p

**DOI:** 10.1042/BSR20190251

**Published:** 2019-04-30

**Authors:** Nan Cheng, Ju Wu, Min Yin, Jian Xu, Yadong Wang, Xi Chen, Zhequn Nie, Jiajun Yin

**Affiliations:** Department of General Surgery, Affiliated Zhongshan Hospital of Dalian University, Dalian City, Liaoning Province 116001, PR China

**Keywords:** hepatocellular carcinoma, lncRNA CASC11, miR-188-5p, survival

## Abstract

It is known that lncRNA CASC11 promotes the development of gastric cancer. Our study was carried out to investigate the possible involvement of ncRNA CASC11 in hepatocellular carcinoma (HCC). In the present study, we found that CASC11 was up-regulated, while miR-188-5p was down-regulated in tumor tissues of HCC patients. CASC11 and miR-188-5p were not affected by HBV and HCV infections. Follow-up study showed that high levels of CASC11 were significantly correlated with poor survival. Expression levels of CASC11 and miR-188-5p were inversely correlated in tumor tissues. CASC11 overexpression mediated the down-regulation of miR-188-5p, while miR-188-5p overexpression failed to affect CASC11 expression. CASC11 overexpression led to promoted, while miR-188-5p overexpression led to inhibited proliferation of cells of HCC cell lines. CASC11 overexpression showed no significant effects on cancer cell migration and invasion. In addition, miR-188-5p overexpression attenuated the enhancing effects of CASC11 overexpression on cancer cell proliferation. Therefore, LncRNA CASC11 promoted cancer cell proliferation in HCC possibly by inhibiting miR-188-5p.

## Introduction

Hepatocellular carcinoma (HCC) is common type of malignant neoplasm in clinical practices, and is the third major cause of deaths amongst cancer patients worldwide [1]. Incidence of HCC is the highest in Asian countries, such as China due to the high prevalence of HBV and HCV infections [2], which are the major causes of many liver diseases including HCC [3]. The development of surgical operations significantly improves the survival of solid tumor patients at early stages, while HCC patients are mostly diagnosed at advanced stages due to its rapid progression and only less than 30% are candidates for surgical operations by the time of initial diagnosis [4], leading to poor overall 5-year survival.

The identification of oncogenes or tumor suppressors in HCC has significantly enriched our understanding on the molecular mechanism of the pathogenesis of HCC [5,6]. However, the limited genetic and epigenetic factors identified so far fail to explain the complex pathogenesis of this disease, indicting the existence of other contributors. In spite of the lack of protein-coding capacity, >200 nts lncRNAs are characterized as critical regulators in cancer biology due to its regulatory roles in gene expression [7]. Regulation of lncRNA expression shows promising potentials in cancer prevention and treatment [8,9]. It has been reported that lncRNA CASC11 promotes colorectal cancer and gastric cancer [10,11]. In a recent study, CASC11 was reported to promote cell migration, invasion, and epithelial–mesenchymal transition in HCC [12]. However, the clinical application of this lncRNA in HCC prognosis is unknown and systemic functional characterization is still need. Our study was therefore carried out to further characterize the function of CASC11 in HCC.

## Materials and methods

### Patients and follow-up

The present study included 68 HCC patients (39 males and 29 females, 38–69 years, 50.3 ± 5.8 years) as research subjects. Those patients were admitted by Affiliated Zhongshan Hospital of Dalian University between March 2010 and May 2013. Inclusion criteria: (1) first time diagnosis; (2) received no therapies before admission; and (3) willing to participate in follow-up. Exclusion criteria: (1) complicated with other clinical disorders; (2) treated before admission; and (3) with history of previous malignancies. Amongst these patients, 26 were HBV-positive, 27 were HCV positive, and 15 were negative for both. Patients, who were positive for both HBV and HCV, were not included in the present study. All patients were followed-up for 5 years to record survivals through outpatient visit or telephone. Patients died of other causes or who were lost during follow-up were not included. All patients signed informed consent. The present study was approved by Affiliated Zhongshan Hospital of Dalian University Ethics Committee.

### Specimens and cell lines

Before therapies, liver biopsy was performed and HCC as well as adjacent non-cancer tissue specimens were collected from each patient. All specimens were confirmed by at least three experienced pathologists.

SNU-398 and SNU-182 HCC cell lines were included in the present study. Cells of both cell lines were bought from ATCC (VA, U.S.A.). Cell culture medium was RPMI 1640 medium with 10% FBS and cell culture conditions were 5% CO_2_ and 37 °C.

### RT-qPCR

TRIzol reagent (Thermo Fisher Scientific, Inc.) was used for total RNA extractions from tissue specimens and *in vitro* cultivated cells. Following reverse transcription performed using Applied Biosystems™ High-Capacity cDNA Reverse Transcription Kit, SYBR^®^ Green Quantitative RT-qPCR Kit (Sigma–Aldrich) was used to prepare qPCR mixture with 18S rRNA as endogenous control to detect the expression of lncRNA CASC11.

mirVana miRNA Isolation kit (Thermo Fisher Scientific, Inc.) was used for miRNA extractions from tissue specimens and *in vitro* cultivated cells. After reverse transcription performed with TaqMan MicroRNA Reverse Transcription Kit (Thermo Fisher Scientific), PCR reaction mixtures were prepared using MystiCq^®^ microRNA^®^ SYBR^®^ Green qPCR ReadyMix™ (Sigma–Aldrich, MO, U.S.A.) to detect miR-188-5p with U6 as endogenous control.

Each experiment included three biological replicates and data normalization was performed using 2^−ΔΔCq^ method.

### Transient transfection

Cell transient transfections were performed using lipofectamine 2000 (11668-019, Invitrogen, Carlsbad, CA, U.S.A.). Negative control miRNA and miR-188-5p mimic were bought from Sigma–Aldrich. CASC11-expression vectors and empty vectors were constructed by Sangon (Shanghai, China). Total 10 nM vectors and 45 nM miRNAs were used in transfections. Two controls, including negative control (negative control miRNA or empty vector transfection) or control (non-transfection) were included. Subsequent experiments were performed at 24 h after transfections.

### Cell proliferation assay

At 24 h after transfection, cells of both SNU-398 and SNU-182 cell lines were harvested and dissolved in RPMI 1640 medium with 10% FBS to prepare single cell suspensions to a final cell density of 5 × 10^4^ cells/ml. Cell suspensions were transferred to a 96-well plate with 0.1 ml per well. Cells were cultured (5% CO_2_ and 37°C). CCK-8 solution (Sigma–Aldrich) was added every 24 h until 96 h with 10 μl per well. After that, cells were cultivated for additional 4 h, followed by the addition of 10 μl DMSO. Finally, OD values (450 nm) were measured to reflect cell proliferation.

### Statistical analysis

Three biological replicates were included in each experiment. Differences amongst multiple groups were analyzed by ANOVA (one-way) and Tukey *t* test. Differences between HCC and non-cancer tissues were analyzed by paired *t* test. Linear regression was used for correlation analysis. The 68 HCC patients were divided into high (n = 33) and low (n = 35) CASC11 level groups using the expression data of CASC11 in HCC tissues according to Youden’s index. Survival curves were plotted using K-M method and compared by log-rank test. Statistically significant level was *P*<0.05.

## Results

### CASC11 and miR-188-5p were dysregulated in HCC tissues

**Figure 1:**
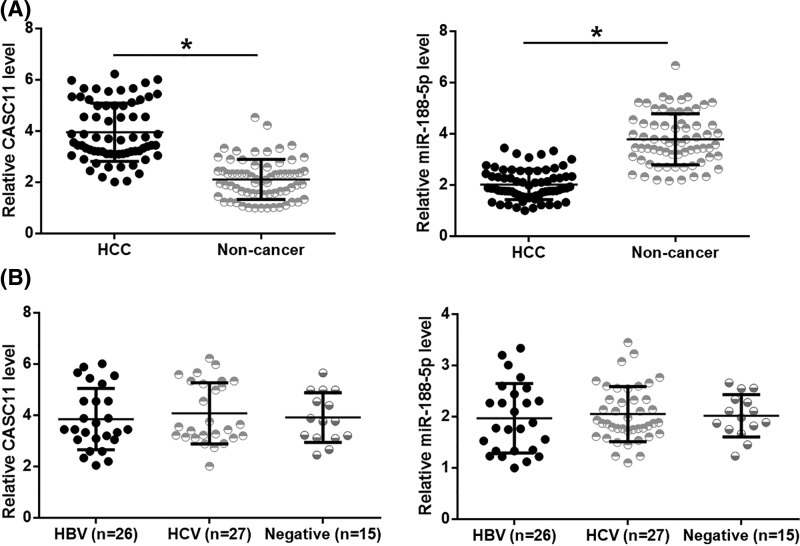
CASC11 and miR-188-5p were dysregulated in HCC tissues RT-qPCR results showed that CASC11 was up-regulated (**A**), while miR-188-5p was down-regulated (**B**) in HCC tissues than in non-cancer tissues of HCC patients. CASC11 (**C**) and miR-188-5p (**D**) expression in HCC tissues were not affected by HBV and HCV infections (*P<0.05).

Expression of CASC11 and miR-188-5p in HCC and adjacent non-cancer tissues was analyzed by performing RT-qPCR experiments. Comparing with adjacent non-cancer tissues, CASC11 was significantly up-regulated (Figure 1A), while miR-188-5p was significantly down-regulated (Figure 1B) in HCC tissues (*P*<0.05). Amongst 68 HCC patients, 26 were HBV-positive, 27 were HCV positive, and 15 were negative for both. Comparisons of CASC11 and miR-188-5p expression levels amongst three groups of patients revealed that expression levels of CASC11 (Figure 1C) and miR-188-5p (Figure 1D) were not significantly different amongst three groups.

### High levels of CASC11 in HCC tissues were significantly correlated with poor survival

**Figure 2:**
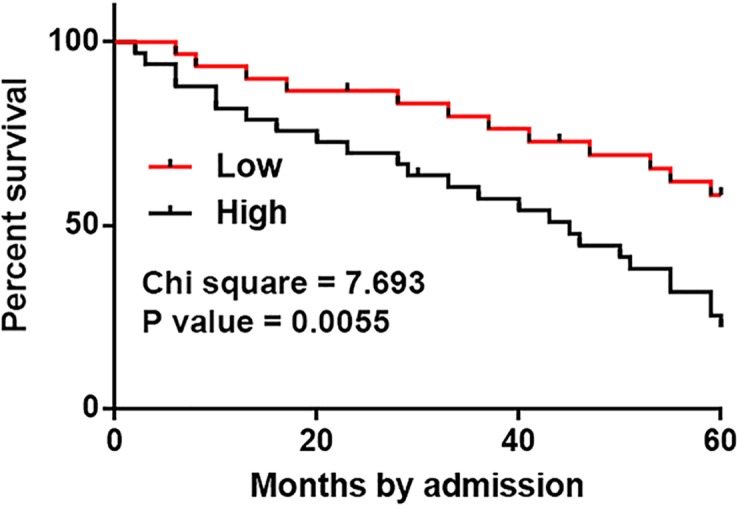
High levels of CASC11 in HCC tissues were significantly correlated with poor survival Survival curve analysis showed that patients in high level group had significantly lower overall survival rate.

The 68 HCC patients were divided into high (*n*=33) and low (*n*=35) CASC11 level groups using the expression data of CASC11 in HCC tissues according to Youden’s index. Survival curves were plotted using K-M method and compared by log-rank test. It was observed that patients in high level group had significantly lower overall survival rate (Figure 2).

### CASC11 and miR-188-5p were inversely correlated in HCC tissues

**Figure 3:**
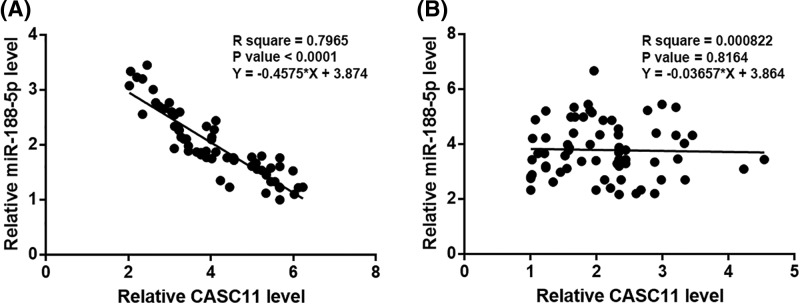
CASC11 and miR-188-5p were inversely correlated in HCC tissues Linear correlation analysis showed that CASC11 and miR-188-5p were significantly and inversely correlated in HCC tissues (**A**), but not in non-cancer tissues (**B**).

Linear correlation was performed to analyze the correlations between expression levels of CASC11 and miR-188-5p. It was observed that CASC11 and miR-188-5p were significantly and inversely correlation in HCC tissues (Figure 3A). In contrast, the correlation between CASC11 and miR-188-5p was not significant in non-cancer tissues (Figure 3B).

### CASC11 overexpression caused down-regulation of miR-188-5p

**Figure 4:**
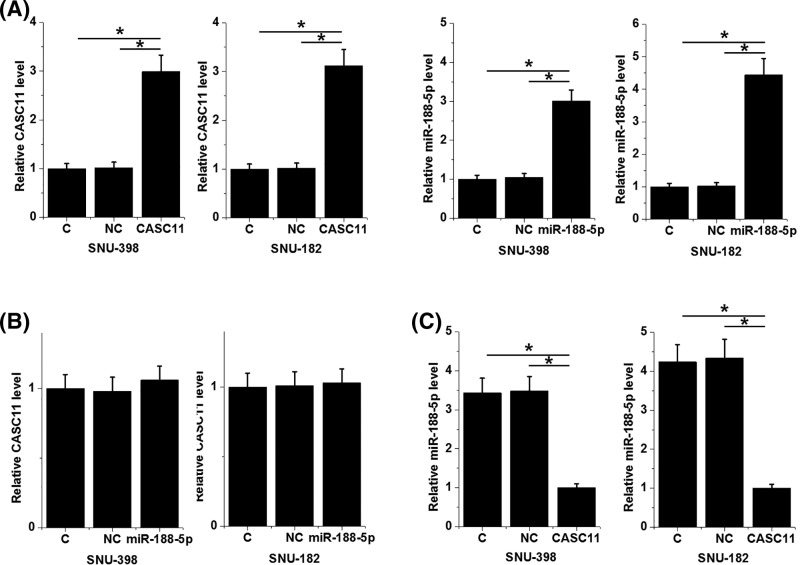
CASC11 overexpression caused down-regulation of miR-188-5p Comparing with two control groups (control, C and negative control, NC), CASC11 and miR-188-5p expression levels were significantly up-regulated in cells of both cell lines (**A**). miR-188-5p overexpression failed to affect CASC11 expression (**B**), while CASC11 overexpression mediated miR-188-5p inhibition (**C**) (*P<0.05).

CASC11-expression vectors and miR-188-5p mimics were transfected into cells of both SNU-398 and SNU-182 cell lines to further analyze the interactions between miR-188-5p and CSAS11. Comparing with two control groups (control, C and negative control, NC), CASC11 and miR-188-5p expression levels were significantly up-regulated in cells of both cell lines at 24 h after transfection (Figure 4A). miR-188-5p overexpression failed to affect CASC11 expression (Figure 4B), while CASC11 overexpression mediated the down-regulation of miR-188-5p (Figure 4C, *P*<0.05).

### CASC11 overexpression promoted HCC cell proliferation through miR-188-5p

**Figure 5:**
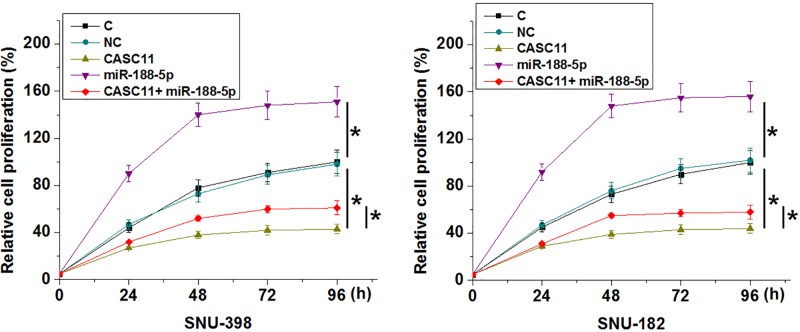
CASC11 overexpression promoted HCC cell proliferation through miR-188-5p Comparing with two control groups (control, C and negative control, NC), CASC11 overexpression led to promoted, while miR-188-5p overexpression led to inhibited proliferation of cells of HCC cell lines. In addition, miR-188-5p overexpression attenuated the enhancing effects of CASC11 overexpression on cancer cell proliferation (*P<0.05).

Comparing to two control groups (control, C and negative control, NC), CASC11 overexpression showed no significant effects on cancer cell migration and invasion (data not shown). In contrast, CASC11 overexpression led to promoted, while miR-188-5p overexpression led to inhibited proliferation of cells of HCC cell lines. In addition, miR-188-5p overexpression attenuated the enhancing effects of CASC11 overexpression on cancer cell proliferation (Figure 5, *P*<0.05).

## Discussion

The present study showed that high expression level of CASC11 was closely correlated with the poor survival of HCC patients, and overexpression of CASC11 may inhibit HCC by reducing the proliferation of HCC cells through the down-regulation of miR-188-5p, which is a tumor suppressor in HCC [13].

Although HBV and HCV infections are the major contributors to the development of HCC, HCC is also closely correlated with other risk factors other than hepatitis infection, such as alcohol or aflatoxin [14]. Pathogenesis of hepatitis-negative HCC is even more complex, and the prevention and treatment are more challengeable [14]. In the present study, we showed that CASC11 was up-regulated in HCC tissues and high expression levels of CASC11 were significantly correlated with the low 5-year overall survival rate. Therefore, detection of the pretreatment level of CASC11 may be used to accurate predict the progression of HCC after treatment and guide the design of individualized follow-up treatment. Interestingly, Han et al. proved that overexpression of CASC11 is closely correlated with the accelerated migration and invasion of HCC cells [12]. However, our study showed that overexpression of CASC11 did not significantly affect the migration and invasion of cells of two HCC cell lines but promoted cell proliferation. This is possible due to the different cell lines used and the heterogeneity of HCC.

MiR-188-5p is tumor suppressive miRNA in HCC [13,15]. MiR-188-5p in HCC suppresses cancer cell proliferation by directly targetting FGF5 [13]. However, the upstream regulator of miR-188-5p in cancer biology is unknown. The present study showed that CASC11 is an upstream inhibitor of miR-188-5p. It is known that lncRNAs may serve as sponge of miRNAs to inhibit their functions [16,17]. However, we performed a local blast with miR-188-5p sequence as query and CASC11 as target, but no promising target site of miR-188-5p was found on the sequence of CASC11. It is known that both CASC11 and miR-188-5p can interact with WNT/β-catenin [10,18]. Therefore, WNT/β-catenin may mediate the interaction between CASC11 and miR-188-5p in HCC. It is worth noting that telomerase and NF-κβ signaling are critical players in HCC [19–21], while Wnt pathway has crosstalk with both telomerase and NF-κβ [22,23]. Therefore, CASC11 may be a component of a complicated gene regulation network involved in HCC.

In conclusion, CASC11 was overexpressed in HCC and may down-regulate tumor suppressive miR-188-5p to promote disease development.

## Availability of data and materials

The analyzed datasets generated during the study are available from the corresponding author on reasonable request.

## Abbreviations

HBV: Hepatitis B virus
HCC: hepatocellular carcinoma
HCV: Hepatitis C virus
RPMI: Roswell Park Memorial Institute
